# The Bergen 4-day treatment for panic disorder: implementation in a rural clinical setting

**DOI:** 10.1186/s12888-023-04812-x

**Published:** 2023-05-01

**Authors:** Thorstein Olsen Eide, Kay Morten Hjelle, Ida Ueland Sætre, Stian Solem, Thorbjørn Olsen, Rolf Olof Sköld, Gerd Kvale, Bjarne Hansen, Kristen Hagen

**Affiliations:** 1 Molde Hospital, Møre og Romsdal Hospital Trust, Molde, Norway; 2 Center for Crisis Psychology, Faculty of Psychology, University of Bergen, Bergen, Norway; 3 Bergen Center for Brain Plasticity, Haukeland University Hospital, Bergen, Norway; 4 Førde Hospital, Førde Hospital Trust, Førde, Norway; 5 Department of Psychology, Norwegian University of Science and Technology, Trondheim, Norway; 6 Department of Clinical Psychology, University of Bergen, Bergen, Norway; 7 Department of Mental Health, Norwegian University of Science and Technology, Trondheim, Norway

**Keywords:** Panic disorder, Intensive treatment, Exposure, B4DT, CBT

## Abstract

**Introduction:**

The Bergen 4-Day Treatment (B4DT) is a concentrated treatment with individually tailored exposure exercises. The format has shown promising results in the treatment of panic disorder.

**Aim:**

The aim of the current study was to investigate the effectiveness of the B4DT in a large sample in a rural clinical setting.

**Method:**

Fifty-eight patients with panic disorder were consecutively included using an open trial design. The primary outcome measure was the Panic Disorder Severity Scale. The Generalized Anxiety Disorder-7 and the Patient Health Questionnaire-9 were used as secondary outcome measures. Assessments were conducted at pretreatment, posttreatment, and 3-month follow-up. Treatment satisfaction was measured at posttreatment using the Client Satisfaction Questionnaire-8.

**Results:**

There was a significant reduction in symptoms of panic disorder from pre- to posttreatment (*d* = 3.36) and from pretreatment to follow-up (*d* = 3.63). At posttreatment and follow-up, 72.4% and 81.0% of patients, respectively, were classified as in remission. Patients reported high treatment satisfaction, and there were significant reductions in symptoms of generalized anxiety and depression.

**Conclusion:**

The results from the current study replicated the findings from previous studies using a larger sample size. The findings indicate that the B4DT is a promising treatment format for panic disorder. The study also demonstrated that the treatment format can be successfully implemented in new rural clinics.

## Introduction

Panic disorder (PD) is characterized by recurring unexpected panic attacks, followed by persistent worry about additional panic attacks or significant maladaptive change in behavior (e.g., avoidance). A panic attack is an abrupt, intense fear or discomfort that reaches a peak within minutes [1]. It is estimated that approximately 28% of the population will experience isolated panic attacks. However, most do not develop PD since they do not experience recurring unexpected attacks. The lifetime prevalence has been estimated to be 3.7% for PD without agoraphobia and 1.1% for PD with agoraphobia [2].

Cognitive behavioral therapy (CBT) has been documented as an effective treatment option for patients with PD with or without agoraphobia [3, 4]. Although research supports the effectiveness of CBT, the treatment is rarely available in general mental healthcare [5]. However, some countries have developed collaborative stepped care treatment as a method for providing evidence-based treatment for anxiety disorders (including panic disorder) in primary care with promising results compared to care as usual [6, 7]. Furthermore, it has been estimated that 40–54% of patients with PD and/or agoraphobia receive treatment consistent with treatment guidelines [2].

Some of the attempts to improve dissemination of CBT for anxiety disorders have included internet-based CBT (iCBT) and blended CBT (combining traditional CBT and iCBT). There has also been growing interest in brief and more intensive treatment for anxiety disorders. These treatments have usually modified traditional CBT by reducing the either number of sessions or the time between sessions, with the goal of making treatment more accessible, efficient, or more cost-effective. Research has suggested that intensified exposure has similar treatment outcomes as ordinary exposure treatment, but intensifying treatment could be associated with quicker response, lower drop-out rates as well as reducing disability days and improving quality of life [8]. Similar findings were reported in a recent meta-analysis, which also added that intensive CBT could be better for reducing comorbid depressive symptoms, but also highlighted that research on intensive CBT formats is understudied [9]. It is also important to note that intensifying exposure treatment could be associated with slightly higher treatment burden and somewhat stronger side effects [10]. A meta-analysis showed that brief, intensive, and concentrated (BIC) CBT for anxiety disorders in children and adolescents had similar treatment effects as standard CBT but lower dropout rates [11].

The Bergen 4 day-treatment (B4DT) is a concentrated form of CBT in which patients receive concentrated exposure treatment in four consecutive days. The format has been demonstrated to be an effective treatment for obsessive-compulsive disorder (OCD) at posttreatment and at 3-month, 1-year, and 4-year follow-ups [12–15]. The B4DT has been adapted for the treatment of PD, and a pilot study demonstrated promising results [16]. Among the participating 29 patients, 72% were in remission and 90% had significantly improved at the 3-month follow-up. The authors found large effect sizes on the primary outcome measure, the Panic Disorder Severity Scale (PDSS) from pre- to posttreatment (*d* = 2.63) and from pretreatment to 3-month follow-up (*d* = 2.76). Patients reported high treatment satisfaction and a significant improvement in secondary outcomes of depression and generalized anxiety symptoms.

A replication study found similar results among 30 patients with PD treated in a new clinical setting in a medium-sized Norwegian city [17]. At posttreatment, 80% were in remission, and the results were stable at the 3-month follow-up (86% in remission). The authors of the replication study found larger effect sizes from pre- to posttreatment (*d =* 4.32) and from pretreatment to 3-month follow-up (*d* = 4.91) than the original study. These treatment effects were also significantly higher than those reported in standard CBT studies [16]. As the results from the two previous studies were promising but had limited sample sizes, it is important to evaluate the B4DT format using a larger sample size. The current study was also conducted in a different clinical setting, that is, a small Norwegian outpatient clinic, in what can be considered among the most rural areas in Norway.

The main aim of this study was therefore to investigate the treatment results after implementing the treatment format in a new rural clinical setting and by recruiting a larger sample. We hypothesized that the treatment would show high remission rates. Furthermore, for benchmarking purposes, the results were compared with the previous two studies on the B4DT for PD and standard CBT for PD.

## Method

### Participants and procedure

The study was a naturalistic quality assessment of treatment outcomes after the implementation of the B4DT format for PD in a rural clinic. Patients (N = 58) who underwent the treatment format and provided written consent to the use of their information from the quality register for research were included. The inclusion of patients took place between 2017 and 2020. Patients were referred to the B4DT team either directly by their general practitioner or by other clinicians in the public mental health care system. All patients were diagnosed pretreatment by a trained and experienced clinician using a structured clinical interview. Patients who met the criteria for a diagnosis of PD with or without agoraphobia were offered treatment. Exclusion criteria were patients who were actively suicidal, psychotic, or bipolar in an unstable phase; who had a primary eating disorder; who demonstrated active substance abuse; or who were not fluent in Norwegian. If the patient wanted the treatment, he or she was granted a place in the next available treatment group. After inclusion, patients received written information about the treatment. Patients were also informed about the structure and focus of the treatment days.

A summary of the sample demographic information is shown in Table 1. The majority of patients had both PD and agoraphobia (89.6%, n = 52). Most of the patients were female (74%), and there was no significant difference in baseline PDSS scores based on gender, *t*(56) = − 0.81, *p* = .493. Half of the patients (53.4%) had previously received treatment for their PD. There was no significant difference in the baseline PDSS scores among patients with previous treatment and those with no treatment, *t*(56) = 1.23, *p* = .233. Most patients had at least one comorbid disorder (74.1%). The most prevalent comorbid disorders were depression (39.7%), generalized anxiety disorder (32.8%), and social anxiety disorder (32.8%). The PDSS scores for patients with comorbidities were not different from those for patients without comorbid disorders, *t*(56) = 1.11, *p* = .271. Several patients (63.7%) used psychotropic medication, and there was no significant difference in PDSS scores among patients using or not using medication, *t*(56) = -0.64, *p* = .526. Twenty-six (44.8%) patients used antidepressants, five (8.6%) used benzodiazepines, 17 (29.3%) used sleeping medication, and one (1.7%) used mood stabilization medication. All patients using medication were informed to maintain the same medication and doses before and during the treatment period. The patients using benzodiazepines (8.5%, n = 5) were encouraged to not use benzodiazepines during and directly after the exposure. All patients adhered to this instruction, and no patients reported withdrawal effects during the treatment period.

**Table 1:** Demographic information (*N* = 58)

Variable	*M* (*SD*)	*n* (%)
Female gender		43 (74.1)
Age	33.6 (12.0)	
Duration of the disorder (years)	9.5 (10.3)	
Previous treatment		31 (53.4)
Marital status		
Single		24 (41.4)
Married or cohabiting		34 (58.6)
Care for children		25 (43.1)
Educational status		
Primary school		9 (15.5)
High school		31 (53.4)
University/college		18 (31.0)
Work status		
Working		34 (58.6)
Studying		5 (8.6)
Unemployed		1 (1.7)
Sick leave		13 (22.4)
Welfare		4 (6.9)
Unspecified leave		1 (1.7)
Comorbidity (any disorder)		43 (74.1)
Depression		23 (39.7)
GAD		19 (32.8)
Social anxiety disorder		19 (32.8)
PTSD		9 (13.8)
OCD		3 (5.2)
Anorexia nervosa		1 (1.7)
Bipolar-II		1 (1.7)
Substance use disorder		1 (1.7)
Alcohol use disorder		1 (1.7)
Psychotropic medication		
Antidepressants		26 (44.8)
Benzodiazepines		5 (8.6)
Sleeping medication		17 (29.3)
Mood stabilization		1 (1.7)

*Note*. GAD = generalized anxiety disorder; PTSD = posttraumatic stress disorder; OCD = obsessive–compulsive disorder

#### Treatment format

The treatment was delivered in groups of 3–6 patients, with a corresponding number of therapists over four consecutive days (21 h on total). The main focus of the 4-day treatment is the “LEaning in Technique” (LET), which emphasizes a shift from avoiding unpleasant symptoms, thoughts and emotions to instead actively approaching situations that elicit anxiety/discomfort.

During the first day, all patients and therapists met for 3–4 h. Thorough psychoeducation covering the core characteristics of PD, maintenance factors, treatment principles, and exposure principles was presented. At the end of the first day, a plan for individual exposure tasks were made for each patient in plenum. The second and third days (approximately 7 h each) focused on individually tailored and therapist-assisted exposure. The group met in the morning, at lunch and at the end of the day to share experiences and report back to the group. The time between these meetings were dedicated to therapist guided in-vivo exposure. The therapy involved in vivo exposure to external situations that the patients either tended to avoid or experienced significant fear in, and interceptive exposure to bodily symptoms that elicited fear in the patients or they had previously attempted to avoid. Patients were encouraged to gain experience in as many relevant settings as possible. Typical exposure tasks often involved physical exercise and hyperventilation to increase psychical arousal, and exposure for agoraphobic situations such as shopping malls, busses, and other situations depending on the individual patient’s avoidance and fear. The focus in all exposure task were practicing the LET-technique where the patients attempted to actively elicit anxiety instead of avoidance or attempting to regulate their anxiety. The patients practiced identifying exactly when they were tempted to initiate avoidance or safety behaviors and then choose to do something that breaks with their previous pattern of behavior (“leaning in”). These moments and choices were taught to the patients as the moments with largest potential for change. Disconfirmation of catastrophic thoughts and a reduction of anxiety during exposure were not a focus of the treatment but rather to find anxiety eliciting situations or thought that could be practiced with the LET-technique. At the end of the second and third days, patients and their therapist made exposure plans for the evening. Patients reported their progress to their therapist by text message. A psychoeducational meeting for relatives or close friends was offered on the third day. The final day (approximately 3–4 h) focused on how to continue training and make the changes an integrated part of patients’ daily lives. The final day further focused on important aspects of relapse prevention. The patients also made an individual exposure plan for the next 3 weeks.

#### Therapists

The treatment was delivered by a team consisting of five therapists. All therapists were certified 4-day treatment therapists. The certification involves participation in a minimum of three groups and hands-on supervision from an experienced 4-day therapist. The group leaders were certified as group leaders, which means that they had participated in a minimum of six groups and had been approved by a 4-day treatment expert. For the current sample, the team consisted of two psychologists, two social workers, and a psychiatric nurse. The therapists’ experience with the treatment of PD varied from six months to 30 years [18].

#### Assessment

Assessment was conducted pretreatment, posttreatment and at three-month follow-up. Patients answered standardized self-report questionnaires online, if the patients did not answer the self-report questionnaires in a pre sett timeframe they received an automated text message reminder. The pretreatment PDSS was conducted by the therapist that did the screening for the treatment. An independent assessor who was not a part of the treatment conducted the posttreatment and follow-up interviews using the PDSS (see below).

#### Clinical measures and diagnostic assessment

The PDSS is a seven-item interview used to assess the severity of PD. The PDSS has excellent interrater reliability and is sensitive to changes after treatment [19]. Scores range from 0 to 28, with higher scores indicating higher severity. Patients without agoraphobia can be considered “slightly ill” with scores of 6–9, “moderately ill” with scores of 10–13 and “markedly ill” with scores of 14 and above. Patients with agoraphobia can be considered “slightly ill” with scores of 8–10, “moderately ill” with scores of 11–15 and “markedly ill” with scores of 16 and above [20].

The Generalized Anxiety Disorder-7 (GAD-7) [21] measures symptoms of generalized anxiety. It is a 7-item questionnaire rated on a four-point Likert scale (0 = not at all, 3 = almost every day). Total scores range from 0 to 21, where a score of 0–4 corresponds to “minimal anxiety”, scores from 5 to 9 correspond to “mild anxiety”, scores from 9 to 14 correspond to “moderate anxiety” and scores from 15 to 21 correspond to “severe anxiety”. The questionnaire has demonstrated good reliability and validity [21].

The Patient Health Questionnaire-9 (PHQ-9) [22] is a questionnaire based on the DSM criteria for depression. The questionnaire has nine items, each rated on a four-point Likert scale (0 = not at all, 3 = almost every day). Total scores range from 0 to 27, where 0–4 corresponds to “none”, 5–9 corresponds to “mild”, 10–14 to moderate, 15–19 corresponds to “moderate severe” and 20 and above is considered “severe”. The questionnaire has good reliability and validity [22].

The Client Satisfaction Questionnaire-8 (CSQ-8) [23] is an 8-item scale rated on a 4-point Likert scale. The CSQ-8 measures the client’s satisfaction with health services. The total score ranges from 8 to 32, with a higher score indicating a higher degree of satisfaction. The questionnaire has shown good psychometric properties [23].

The Mini International Neuropsychiatric Interview (MINI) [24] was used to assess PD diagnosis and comorbid disorders before inclusion. The MINI is a structured interview for axis-1 DSM-IV disorders. The interview was administered by an experienced clinician. The Norwegian version has been found to have good psychometric properties [25].

### Statistical analysis

The dataset had relatively few instances of missing data. In the PDSS, 5.17% of data were missing from the different assessment points. Five patients (8.62%) had missing responses at posttreatment, and four patients (6.89%) had missing responses at the 3-month follow-up. For the self-report measurements, there were a total of 10.6% missing responses across all assessment points. Missing data were replaced using the expectation maximization (EM) method of SPSS version 27. When less than 25% of the dataset is missing and data are missing at random, which was the case in this data set (Little’s MCAR test; *x*^2^ (163) = 163,237, *p* = .480), EM is an effective method for replacing missing data [26]. Statistical analysis was conducted using data from all 58 patients. Repeated measures ANOVA for the PDSS, GAD-7, and PHQ-9 was conducted, and the Greenhouse‒Geisser correction was used in cases when Mauchly’s test of sphericity was significant.

Response and remission were defined according to the Furukawa et al. [20] criteria in which a reduction of 40% on the PDSS is defined as treatment response. Patients were considered in remission if they post-treatment were classified in the “borderline ill” category or better, which is 5 for PD and 7 for PD with agoraphobia.

## Results

### Clinical outcome

Patients showed a significant and large reduction in their PDSS scores from pre- to posttreatment and a significant and moderate reduction from posttreatment to the 3-month follow-up (see Table 2). Mauchly’s test was significant (*p* = .031); therefore, Greenhouse–Geisser correction was applied. The repeated-measures ANOVA indicated a significant change in PD symptoms, *F*(1.791, 102.095) = 303.22, *p* < .001, partial *µ*^2^ = 0.842. There was a significant reduction in symptoms from pretreatment to posttreatment (p < .001, *d* = 3.36) and from pretreatment to follow-up (*p* < .001, *d* = 3.64). There was also a significant reduction in PD symptoms from posttreatment to follow-up (*p* = .001, *d* = 0.61).

**Table 2:** Results (*M* and *SD*) for the primary and secondary outcome measures (*N* = 58)

Variable	Pre	Post	Follow-up	Cohen’s *d*	
				Pre-post	Pre-follow-up
PDSS	16.10 (3.90)	4.72 (2.77)	2.81 (3.38)	3.36	3.64
GAD-7	12.31 (4.09)	6.34 (4.33)	5.50 (4.05)	1.41	1.67
PHQ-9	12.59 (5.87)	6.77 (4.98)	6.95 (5.40)	1.06	1.00
CSQ-8		29.40 (2.25)			

*Note*. PDDS, Panic Disorder Severity Scale; GAD-7, Generalized Anxiety Disorder-7; PHQ-9, Patient Health Questionnaire-9. CSQ-8 Client Satisfaction Questionnaire-8 d = Cohen’s *d = (M*_*pre*_*– M*_*post*_*)/SD*_*pooled*_

There was also a significant reduction in symptoms of generalized anxiety, *F*(1.749, 99.709) = 64.99, *p* < .001, partial *µ*^2^ = 0.533. There was a significant change from pretreatment to posttreatment (p < .001, *d* = 1.41), as was the change from pretreatment to follow-up (*p* < .001, *d* = 1.67). There was no change from posttreatment to follow-up (*p* = .282, *d* = 0.20). Symptoms of depression also showed a significant reduction, *F*(1.732, 98.707) = 51.71, *p* < .001, partial *µ*^2^ = 0.476. The reduction in symptoms of depression was significant from pretreatment to posttreatment (p < .001, *d* = 1.06), and the reduction from pretreatment to follow-up was significant (*p* < .001, *d* = 1.00). There was no significant change in symptoms of depression from posttreatment to follow-up (*p* = .852, *d* = 0.03).

Patients reported high satisfaction with the treatment. The mean score on the CSQ-8 was 29.40 (*SD* = 2.25). The scores ranged from 23 to 32, and the most common scores were 32 (24.1%) and 31 (15.5%).

### Responses and remission

At posttreatment, 86.2% of participants were classified as treatment responders, and 72.4% of patients were considered in remission. At follow-up, 81.0% of patients were classified as in remission, and 81.0% were responders (see Table 3). Based on the criteria for the PDSS at posttreatment, 46.6% were defined as “very much improved”, 39.7% as “much improved”, 1.7% as “minimally improved”, and 3.4% as “not improved. At follow-up, 67.2% of patients were defined as “very much improved”, 19% as “much improved”, 5.2% as “minimally improved” and 1.7% as “not improved”.

**Table 3:** Status at follow-up based on criteria from Furukawa et al. [20]

	Posttreatment, *N* (%)	Follow-up, *N* (%)
Response	50 (86.2%)	47 (81.0%)
Remission	42 (72.4%)	47 (81.0%)
Very much improved (75–100%)	27 (46.6%)	39 (67.2%)
Much improved (40–74%)	23 (39.7%)	11 (19.0%)
Minimally improved (10–39%)	1 (1.7%)	3 (5.2%)
No improvement (0–10%)	2 (3.4%)	1 (1.7%)
Missing data	5 (8.6%)	4 (6.9%)

### Comparisons with previous studies on the B4DT for PD

Compared to the pilot study of the B4DT for PD, the current study did not differ significantly in terms of the pretreatment PDSS scores (*p* = .72, *d* = 0.07) or posttreatment scores (*p* = .41, *d* = 0.17), but the current study showed significantly lower PDSS scores at the 3-month follow-up (*p* = .012, *d* = 0.57) [16]. Compared to the replication study, the current study showed significantly lower PDSS scores at pretreatment (*p* < .001, *d* = 1.01). The results of the current study did not differ significantly from those of the replication study at posttreatment (*p* = .62, *d* = 0.10) or at the 3-month follow-up (*p* = .82, *d* = 0.05) [17].

Regarding treatment satisfaction, there was no significant difference between the current study and the pilot study (*p* = .16, *d* = 0.29) [16]. There was a significantly lower score in treatment satisfaction in this study compared to that in the replication study (*p* = .005, *d* = 0.66) [17].

### Comparison to standard CBT for PD

The pooled effect of CBT studies using the PDSS as an outcome measure were used to benchmark the results from this study to standard CBT for PD (see Table 4). A search using the PsycINFO database for face-to-face CBT studies of PD with or without agoraphobia using PDSS identified six studies [27–32]. Compared to the patients receiving standard CBT, the patients in the current study had significantly higher PDSS scores at pretreatment (*p* = .001, *d* = 0.46) and significantly lower PDSS scores at posttreatment (*p* < .001, *d* = 0.67) and at the 3-month follow-up (*p* < .001, *d* = 0.55).

## Discussion

This is the second replication study of the B4DT for PD, and the results further strengthen the finding that the B4DT can be replicated successfully in other clinical settings using other therapists and as a part of routine clinical care in a rural clinic. The study had large effect sizes from pre- to posttreatment and from pretreatment to follow-up. This study had twice the sample size of previous studies on the B4DT for PD, indicating that the treatment results are maintained when the treatment is implemented as part of ordinary care. Compared to the pilot study, this study found significantly lower PDSS scores at follow-up, but there was no significant difference between the results of this study and those of the replication study at posttreatment or at the 3-month follow-up.

The results of this study are in line with promising findings of temporally intensified exposure for anxiety disorders by Pittig et al. [8] who included different anxiety disorders and used other outcome measures. Both studies found large within effect sizes for primary outcomes but also for secondary outcomes such as depression. It is important to note that comparisons with other studies are difficult for several methodological reasons, so interpretations should be made cautiously.

Compared to patients receiving standard CBT, patients in this study had significantly higher scores pretreatment (see Table 4). This is in line with previous studies on the B4DT [16, 17] indicating that patients offered the B4DT may generally have higher PD severity than the average PD patient in other studies. Compared with standard CBT studies, the current study also found significantly lower scores at posttreatment and at the 3-month follow-up, which is also in line with previous studies on the B4DT for PD [16, 17]. The comparison with standard CBT should be interpreted with caution, as the effect size can differ due to different study designs. Higher pretreatment scores might also have contributed to the higher effect sizes found in the B4DT.

**Table 4:** Comparison between current study, earlier studies on B4DT and standard CBT

Time	Current study	Hansen et al. (16)	*p*	*d*	Iversen et al. (17)	*p*	*d*	Standard CBT (27–32)	*p*	*d*
Pre	16.10 (3.90)	15.79 (3.97)	0.72	0.07	19.83 (3.44)	< 0.001	1.01	14.39 (3.48)	0.001	0.46
Post	4.72 (2.77)	5.34 (4.22)	0.41	0.17	4.37 (3.72)	0.62	0.10	7.03 (3.95)	< 0.001	0.67
Follow-up	2.81 (3.38)	4.82 (3.65)	0.01	0.57	2.62 (3.57)	0.82	0.05	4.88 (4.00)	< 0.001	0.55

*Note*. PDDS, Panic Disorder Severity Scale; d = Cohen’s *d = (M*_*pre*_*– M*_*post*_*)/SD*_*pooled*_

The secondary outcome measurements also demonstrated a significant reduction (large effect sizes) in the symptoms of depression (PHQ-9) and anxiety (GAD-7) from pretreatment to posttreatment and from pretreatment to 3-month follow-up. The large reduction in primary outcome and secondary outcome measures is in line with previous research on the B4DT for PD [16, 17] and with results from the B4DT for OCD [13–15, 33]. The large reduction in secondary outcome measures for depression found in the current sample and previous research on the B4DT is in line with meta-analytic findings from Remmerswaal et al. [9].

The treatment was well accepted by the patients, and there were no dropouts from the patients in this study, while 18% of patients receiving standard CBT dropped out [16]. This might be because of the concentrated treatment format, which has previously been demonstrated to have a significantly lower dropout rate than regular treatment [11]. The patients also scored high on treatment satisfaction as measured by the CSQ-8.

The study has several limitations, as it was an open trial study with no control conditions. However, PD can be considered a chronic disorder without treatment [34], with little change in the waiting list conditions [35, 36]. The study was conducted as a part of ordinary clinical care, and there were no video recordings to check for adherence. This study demonstrated that the results from the treatment format are promising, but they need to be replicated in randomized controlled studies and compared to other active treatments. This is the second study demonstrating that the B4DT can be successfully implemented for PD at new treatment sites, and there should be further studies replicating the findings in new sites and other cultural contexts. As there is a lack of long-term follow-up studies on CBT for PD [37], the long-term effectiveness of this treatment format needs further examination.

## Conclusion

The Bergen 4-Day treatment can be successfully implemented in new treatment sites and is a promising treatment format for disseminating evidence-based treatment for PD to new treatment sites. In addition to a significant reduction in panic symptoms, the secondary symptoms of depression and generalized anxiety demonstrated a significant reduction. The treatment had no patient dropout and a high degree of patient satisfaction.

## Data Availability

The dataset for the study is available from the first author upon reasonable request.
